# PHYSICAL ACTIVITY, SEDENTARY BEHAVIORS AND MYOSTEATOSIS IN AFRICAN CARIBBEANS

**DOI:** 10.1093/geroni/igad104.3526

**Published:** 2023-12-21

**Authors:** Lily Bessette, Ryan Cvejkus, Adrianna Acevedo-Fontanez, Allison Kuipers, Joseph Zmuda, Victor Wheeler, Iva Miljkovic

**Affiliations:** University of Pittsburgh, Pittsburgh, Pennsylvania, United States; University of Pittsburgh, Pittsburgh, Pennsylvania, United States; University of Pittsburgh, Pittsburgh, Pennsylvania, United States; University of Pittsburgh, Pittsburgh, Pennsylvania, United States; University of Pittsburgh, Pittsburgh, Pennsylvania, United States; Scarborough General Hospital, Tobago, Tobago, Trinidad and Tobago; University of Pittsburgh, Pittsburgh, Pennsylvania, United States

## Abstract

Skeletal muscle fat infiltration (myosteatosis) leads to poorer muscle quality and function with aging, and is emerging as a predictor of skeletal muscle strength, mobility, and metabolic disorders. Few studies investigated the association of physical activity (PA) and sedentary behavior (SB) with myosteatosis, especially in African Caribbeans, a vastly under-studied, rapidly growing population. We examined the associations of objectively measured PA and SB (Bodymedia SenseWear Armbands) worn over 7 days with calf muscle density (mg/cm3), a marker of intra-muscular fat (Stratec XCT-2000), in older adult African Caribbean men (N=355, mean age 62 years) and women (N=682, mean age 59 years) of the Tobago Health Study. Compared to men, women were younger with higher BMI, more SB, less light (LPA) and moderate to vigorous PA (MVPA), and lower muscle density (p< 0.05, age-adjusted). Meeting MVPA guidelines (150 min MVPA/week) was associated with greater muscle density in obese men and women (p=0.03, p=0.09 respectively, age-adjusted). Adjusting for age, BMI, smoking, diabetes, hypertension, and lipid-lowering treatment, we assessed sex stratified partial spearman correlations between PA and SB and muscle density. MVPA was more strongly correlated with greater muscle density in men (r=0.15, p=0.01) than in women (r=0.07, p=0.09), while SB was associated with lower muscle density in women (r=-0.08, p=.044), but not significantly in men (r=-0.10, p=0.12). Our novel findings indicate that the association between PA and SB with myosteatosis may differ by sex. These findings may have important implications for sex specific activity guidelines related to skeletal muscle health in African Caribbeans.

